# Assessment of differences between DNA content of cell-cultured and freely suspended oocysts of *Cryptosporidium parvum* and their suitability as DNA standards in qPCR

**DOI:** 10.1186/s13071-019-3851-7

**Published:** 2019-12-19

**Authors:** Ian D. Woolsey, Berit Blomstrand, Øivind Øines, Heidi L. Enemark

**Affiliations:** 1 Department of Animal Health and Food Safety, Norwegian Veterinary Institute, Ullevålsveien 68, 0454 Oslo, Norway; 2 Norwegian Centre for Organic Agriculture, Gunnars veg 6, 6630 Tingvoll, Norway

**Keywords:** *Cryptosporidium*, HCT-8, Cell culture, qPCR

## Abstract

**Background:**

Although more modern methods are available, quantitative PCR (qPCR) is reproducible, sensitive and specific with instruments and expertise readily available in many laboratories. As such, the use of qPCR in *Cryptosporidium* research is well established and still widely used by researchers globally. This method depends upon the generation of standards at different concentrations to generate standard curves subsequently used for the quantification of DNA.

**Methods:**

We assessed four types of DNA template used to generate standard curves in drug screening studies involving *Cryptosporidium* spp.: (i) serially diluted *Cryptosporidium parvum* oocysts (10^6^–1); (ii) diluted template DNA from pure oocysts (×10–×10^6^ dilution of 10^6^ oocyst DNA template); (iii) oocysts incubated in human ileocecal adenocarcinoma (HCT-8) cells (10^5^–1 and 5 × 10^4^–50); and (iv) diluted DNA template (5 × 10^4^) from cell culture incubated parasites (×10–×1000).

**Results:**

Serial dilutions of both cell culture and pure oocyst suspension DNA template yielded better linearity than cell culture derived standards, with dilutions of 10^6^ oocysts exhibiting similar quantification cycle (Cq) values to those obtained from DNA template dilutions of 10^6^ oocysts. In contrast, cell culture incubated oocysts demonstrated significantly higher DNA content than equivalent freely suspended oocysts and diluted DNA template from both cell culture derived and freely suspended oocysts across numerous concentrations.

**Conclusions:**

For many studies involving *Cryptosporidium*, only relative DNA content is required and as such, the superior linearity afforded by freely suspended oocysts and diluted DNA template (from either cell culture derived standards or freely suspended oocysts) will allow for more accurate relative quantification in each assay. Parasite division in the cell culture standards likely explains the higher DNA content found. These standards, therefore, have the potential to more accurately reflect DNA content in cell culture assays, and despite more modern methods available for absolute quantification, i.e. droplet digital PCR (ddPCR), the ubiquity of qPCR for the foreseeable future encourages further investigation into the reduced linearity observed in these standards such as varying oocyst seeding density, non-linear growth rates and assay efficiency.
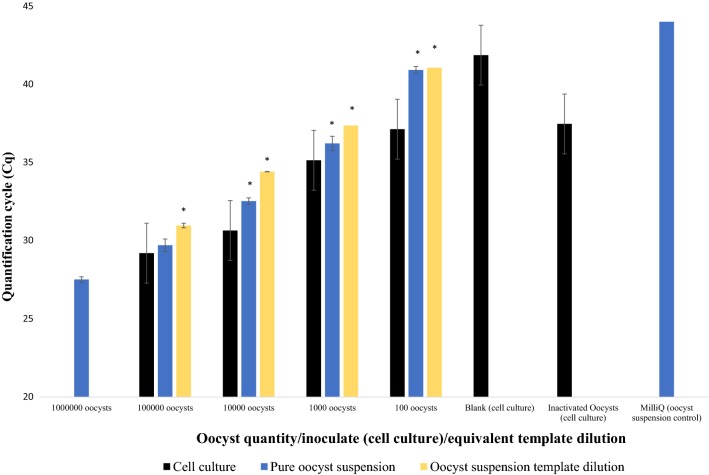

## Background

Protozoan parasites of the genus *Cryptosporidium* are a significant cause of enteric disease in humans and animals globally [1]. In humans, cryptosporidiosis is considered the second leading cause of diarrhoea and death in children after rotavirus [2]. *Cryptosporidium* spp. is ranked the fifth food-borne parasite in importance in Europe after *Echinococcus* spp. (*E. granulosus* and *E. multilocularis*), *Toxoplasma gondii*, and *Trichinella spiralis* based on the FAO/WHO criteria [3]. *Cryptosporidium* spp. are transmitted *via* the faecal-oral route and oocysts may remain viable in the environment for long periods of time [4]. Oocysts are resistant to commonly used disinfectants and no vaccines are available [5, 6]. At present, treatment options are insufficient and vary between countries, with only one drug approved for use, nitazoxanide (Alinia^®^, Romark L.C., Tampa, USA) in humans and halofuginone lactate (Halocur^®^, Intervet Productions S.A., Igovilles, France; Halagon^®^, Divasa-Farmavic, S.A., Barcelona, Spain; Kriptazen^®^, Virbac, Carros, France), and paromomycin (Parofor^®^, Huvepharma NV, Sofia, Bulgaria) in calves, respectively. However, none of the drugs are able to completely prevent or cure the disease [1, 7].

Numerous methods have been employed to detect the presence of *Cryptosporidium* oocysts in stool or water samples for diagnostic purposes and water quality evaluations, to assess their infectivity, and to screen potential compounds for anti-*Cryptosporidium* efficacy (e.g. [8, 9]). The use of cell culture for the assessment of *Cryptosporidium* growth is widely established and in addition to determining the presence and varying infectivity of oocysts, this experimental model allows the screening of candidate compounds against *Cryptosporidium* spp. more economically than *in vivo* studies [10, 11]. Cell culture approaches allow different parasite stages to be exposed to various compounds and to different concentrations *in vitro* so that their relative growth compared to an unexposed control may be determined (e.g. [12, 13]). This method has been supplemented by various methods for quantification of the parasite, including the use of immunofluorescence [12, 14] and conventional polymerase chain reaction (PCR) [15, 16]. Successful development of *C. parvum* was first reported in cell culture by Current & Haynes in 1984 [17]. Although many improvements have been made since then, large-scale parasite reproduction in cultured systems is yet to be achieved. Oocyst production in cell lines has been described but much information regarding the development of the different parasite stages remain difficult to interpret. Due to these problems, complimentary studies are often performed and mouse models are still considered the gold standard for evaluation of infectivity of *Cryptosporidium* oocysts. However, cell culture systems can still provide valuable information regarding *Cryptosporidium* biology much more quickly and affordably than animal models [18].

Quantitative PCR (qPCR) represents an important improvement over conventional PCR [19] with this method allowing for the quantification of DNA target in real time and has proven to be reproducible, sensitive and specific [20]. Real-time instruments are often already available in laboratories as a wide variety of molecular methods have been developed on these platforms, and as qPCR reactions can be easier to automate, this also allows for higher throughput of samples. One key drawback of this platform, when used for quantification, is the need for several standards at different concentrations (representing the dynamic range of the assay) to ensure generation of standard curves to allow the subsequent quantification of DNA in the samples [21], and these need to be included in each PCR run to address inherent variability and performance of the PCR. These small, random and technical variations may arise due to minor differences in running conditions, instrument reproducibility, variation in the samples investigated or variations of concentrations of components in the PCR reactions.

Methods employed to make controls that are used as templates to establish standard curves for *Cryptosporidium* qPCR quantification, vary from serial dilutions of template DNA extracted from a known stock concentration of oocysts [21–23], serial dilution of a known stock concentration of oocysts and subsequent extraction [24, 25], template prepared from inoculation of serial diluted oocyst suspensions into cell culture [26–28], the serial dilution of extracted template DNA from a known quantity of oocysts inoculated onto cell culture [27], or serial dilution of cloned amplification targets in plasmids [27, 29].

*Cryptosporidium parvum* is a proliferative parasite, undergoing both asexual and sexual division with infective sporozoites developing into trophozoites, meronts and 6–8 merozoites each (and therefore contain approximately 6–8 nuclei each) [30], each of which differ in volume compared with that of the initial invading sporozoite [13, 31]. Thus, the applicability of these different standards and careful attention to how they are prepared is necessary. For investigations aiming to merely identify and quantify oocysts from stool or water samples, it would be logical to use standards that are prepared from serial dilutions of a known quantity of oocysts (or serial dilutions of extracted template DNA from a known quantity of oocysts), and this approach has been reported from several studies [21, 25]. Similarly, serial dilutions of a known quantity of oocysts incubated in cell culture, under similar conditions would logically improve the assessment of the presence of any comparative inhibitory potential of these compounds against *Cryptosporidium* oocysts. Such disinfection/inactivation studies utilising cell culture have previously been reported [10, 24, 26, 28].

Some studies utilise serial dilutions of free oocysts as standards for parasite samples after being grown in cell culture, or for parasite development in cell-free culture [22–24]. As the *relative*, rather than *absolute* quantification, is relevant for compound screening and disinfection studies as only significant effects on parasites are relevant, the use of such standards could still be a feasible alternative. High variation is inherent within *Cryptosporidium* cell culture assays [9] and could make any attempts of generating precise and uniform standards for accurate parasite count challenging.

In qPCR reactions, the quantity of a specific DNA target in a sample is calculated from the observed quantification cycle (Cq) value and compared to the quantification cycle of known standards. It is important that the sample DNA is run in parallel under identical conditions with the group of controls. The accuracy of the data extrapolated from these experiments is therefore highly dependent on both the accuracy of the DNA standard quantification, and the quality of the standard curve generated [32]

Recently, digital droplet PCR (ddPCR) has been a method that somewhat overcame the variety of errors introduced when making these standard curves [21]. In ddPCR, partitioning of the samples into thousands of single droplets containing mostly one or no target DNA molecules, and subsequently performing a PCR reaction in this droplet, can eliminate the errors introduced by using standard curves [21]. Although the ddPCR approach allows highly accurate concentration calculations, the higher costs associated with the additional purchase of special ddPCR instruments and reagents could be considerable for a laboratory. Additionally, qPCR enables increased throughput compared to ddPCR platforms [21] and it is therefore likely qPCR will remain a widely relevant and useful platform in the future. In an effort to assess the appropriateness of these standards in future experiments screening for candidate compounds against *Cryptosporidium* spp. *in vitro*, we determined the differences in parasite DNA content using qPCR and data from Cq-values from four different standards: (i) serially diluted *Cryptosporidium parvum* oocysts; (ii) diluted template DNA from pure oocysts; (iii) equivalent concentrations of oocysts incubated in cell culture; and (iv) diluted DNA template from cell culture incubated parasites.

## Methods

### Parasite material

*Cryptosporidium parvum* oocysts (Iowa strain) were purchased from Bunch Grass Farm (ID, Deary, USA) and stored in 50 ml phosphate-buffered saline (PBS) with penicillin (1000 IU) and streptomycin (1000 μg) at 2 × 10^7^/ml (stock solution). According to the manufacturer, the oocysts were shed 14 days prior to delivery to the institute. The oocyst viability was assessed to be 82% *via* 4ʹ,6-diamidino-2-phenylindole/propidium iodide (DAPI/PI) stained oocysts in wet mounts immediately prior to inoculation of the first assay plate [33, 34]. Oocyst morphology appeared normal, with a very low percentage (5%) of ‘ghost’ oocysts (oocysts with no internal content). Oocysts were approximately 3 months-old when the first assay was conducted with all assays conducted within 10 days. Oocysts for inoculation of the plates were taken directly from the stock solution and prior to performing the assays, the oocyst concentration was determined by diluting 10 μl stock solution with 990 μl PBS and counting three repeats after staining with Crypt-a-Glo (Waterborne Inc, New Orleans, LA, USA).

### HCT-8 cell culture

Human colon adenocarcinoma (HCT-8) cells (European collection of authenticated cell cultures (ECACC), Salisbury, UK) were cultured in maintenance medium (RPMI-1640, Biowest, Nuaillé, France) supplemented with horse serum (5% v/v), foetal bovine serum (FBS) (5% v/v), 1 mM sodium pyruvate and an antibiotic/antimycotic solution (penicillin (100 U/ml), streptomycin (100 μg/ml) and amphotericin B (0.25 μg/ml)) (all Sigma-Aldrich, St. Louis, MO, USA). Cell sub-cultivation was performed twice per week with trypsin/ethylenediaminetetraacetic acid (EDTA) (Sigma-Aldrich). Cells were incubated in 5% CO_2_ at 37 °C.

### Parasite inoculation onto cell monolayer

HCT-8 cells were seeded onto Nunclon^®^ 96-well plates (Sigma-Aldrich) (2 × 10^5^ cells/well) and incubated in maintenance medium for 24 h until they reached confluence. Prior to infection of the cell monolayer, the oocysts were pre-treated as described by Slifko et al. [35]. Briefly, one part oocyst stock solution (2 × 10^6^ oocysts) was suspended in 1 part bleach solution, and 8 parts MilliQ water (1107.2 ml MilliQ water, 138.4 µl oocyst suspension and 138.4 μl 5.25% sodium hypochlorite solution) for 10 min. The suspension was subsequently centrifuged at 4000×*g* for 4 min at 4 °C and the supernatant aspirated. The oocysts were then washed in 14 ml MilliQ water and centrifuged again (4000×*g*, 4 min, 4 °C), aspirated down to 200 μl and re-suspended in 1.8 ml maintenance medium pre-warmed to 37 °C to achieve a concentration of 10^6^ oocysts/ml. The oocyst suspension was vortexed (10 s) and 100 μl (100,000 oocysts) was subsequently diluted 10-fold to achieve 6 suspensions (10^5^, 10^4^, 10^3^, 10^2^, 10^1^ and 1 oocysts/100 μl). Of each suspension, 100 μl was inoculated onto the cell monolayers (×5 repeats of each suspension) with a multi-channel pipette and incubated for 4 h (37 °C, 5% CO_2_). Maintenance medium was added to 5 blank wells with cell monolayers to control for potential oocyst contamination and inactivated oocysts in five replicates (5 × 10^5^) (incubated at 70 °C, 30 min) as a control for the washing steps. After incubation the oocysts suspensions and maintenance media were aspirated and washed once for 5 min (37 °C PBS) and 100 μl fresh maintenance medium (1% dimethyl sulfoxide, DMSO) was added to the monolayers. Maintenance medium (100 µl, 1% DMSO) was added to 2 wells containing no cells as a further control for contamination. Plates were subsequently incubated for 44 h. Oocyst inoculations were performed twice, on two separate plates, A and B. HCT-8 cells were on their 4th passage prior to use in the assays.

### Oocyst serial dilution

Stock oocysts (1 ml, 2 × 10^7^/ml) were centrifuged at 4000×*g* for 5 min and the supernatant aspirated down to 200 μl to yield an oocyst suspension of 2 × 10^7^/200 μl. This suspension was vortexed (10 s) and diluted 10-fold (MilliQ water) ×6 to achieve 7 suspensions: 10^6^,10^5^,10^4^, 10^3^, 10^2^, 10^1^ and 1 oocysts/10 μl in 1.5 ml Eppendorf tubes. This was performed twice, yielding two separate serial dilutions of oocyst suspensions. For each dilution, a 1.5 ml Eppendorf tube containing 200 μl of MillQ was prepared in parallel as a negative control.

### DNA extraction

After incubation for 44 h, the maintenance medium was aspirated and a water-soluble tetrazolium (WST)-1 assay (Roche, Basel, Switzerland) performed to assess cell viability as per manufacturer’s instructions. Briefly, 100 μl 10% v/v WST-1 solution in maintenance medium was added to each well and plates incubated for 30 min (37 °C, 5% CO_2_). Subsequently, optical density (OD) was read at 450 nm. Cells were then washed 3 × 5 min (100 μl, 37 °C PBS) prior to cell lysis. Proteinase K (20 μl) and animal tissue lysis (ATL) buffer (180 μl) (Qiagen, Hilden, Germany) were added to each well and incubated (56 °C) for 90 min. All well contents were aspirated into 1.5 ml Eppendorf tubes. For pure oocyst suspensions, Proteinase K and ATL buffer were added directly into 1.5 ml Eppendorf tubes containing 10 μl oocyst suspension. DNA was extracted using QiaCube (DNeasy® Blood & Tissue Kit, Qiagen) with the tissues and rodent tails protocol (elution volume 200 μl). DNA template from 10^6^ of the pure oocyst suspension was then diluted 10-fold (6 dilutions corresponding to equivalent pure oocyst suspension dilutions) with dH_2_O (nuclease-free water-Integrated DNA Technologies, Coralville, IA, USA).

### qPCR assay

**Fig. 1:**
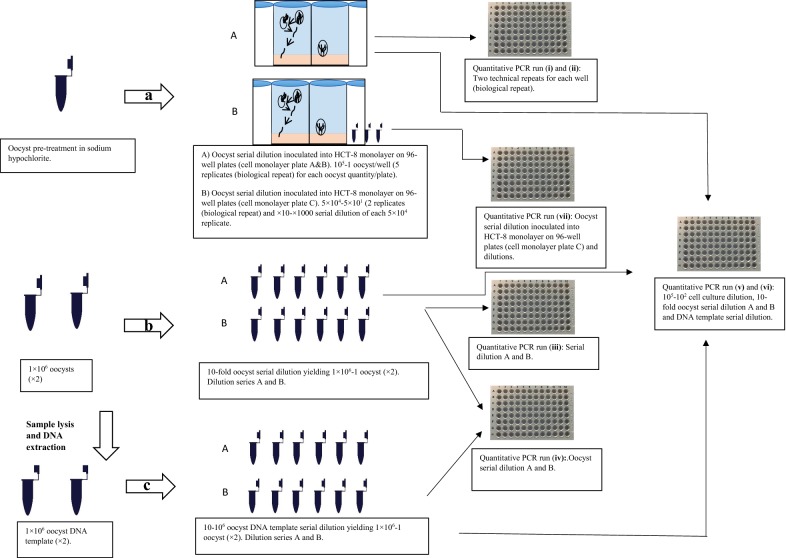
Schematic diagram for each of the three methods for producing standard DNA templates. Ten-fold dilutions of *Cryptosporidium parvum* oocysts inoculated onto cell culture (**a)**, ten-fold dilutions of pure oocyst suspensions (**b**) and ten-10^6^ dilutions of 10^6^ *C. parvum* oocyst extracted DNA template (**c**). Colour illustration in figure adapted from [12] and used with permission

Real-time PCR was carried out using the *18S* primers described previously by Morgan et al. [36] (Cryp18S_Frt 5ʹ-AGT GAC AAG AAA TAA CAA TAC AGG-3ʹ and Cryp18S_Rrt 5ʹ-CCT GCT TTA AGC ACT CTA ATT TTC-3ʹ) with the hydrolysis probe containing an internal ZEN™ quencher to lower background and increase signal detection (5ʹ-/56-FAM/ACC AGA CTT/ZEN/GCC CTC C/3IABkFQ/-3ʹ). Probe design was from Keegan et al. [23], and was based on a conserved eukaryotic sequence first used by Amman et al. [37] both supplied by Integrated DNA Technologies (Coralville). PCR reactions were carried out in a total volume of 25 μl comprising of 3 μl forward and reverse primers, 0.6 μl probe, 12.5 μl 2× Brilliant III Fast MasterMix (Agilent Technologies, Santa Clara, CA, USA), 5.4 μl dH_2_O (nuclease-free water, Integrated DNA Technologies) and 0.5 μl DNA template. Two technical repeats were run for each sample in all assays. Six assays were run on the BIORAD CFX real-time instrument (BioRad, Hercules, CA, USA) using 96-well clear bottomed qPCR plates (BioRad). Six different qPCR runs were prepared: (i) and (ii) oocyst monolayer inoculations (cell monolayer plate A on qPCR Plate (i), and cell monolayer plate B on qPCR Plate (ii); (iii) stock oocyst serial dilution A and B; (iv) oocyst stock dilution A and B with their respective DNA template dilutions; and (v) and (vi) 3 biological repeats from the monolayer inoculations at the four highest inoculation concentrations (10^5^, 10^4^, 10^3^ and 10^2^), oocyst stock dilutions at the 5 highest suspension concentrations (10^6^, 10^5^, 10^4^, 10^3^ and 10^2^) and their respective DNA template dilutions. It was subsequently determined that differences between cell culture incubated oocysts and equivalent DNA template dilutions of the highest concentration would be valuable. DNA template from a separate oocyst titration study (cell monolayer plate C, 5 × 10^4^–50, 2 biological repeats per oocyst concentration) were run on Plate (vii) alongside 5 × 10^4^ ten-fold diluted template (×10–×1000, one dilution series for each biological repeat) (Fig. 1). This later titration study (cell monolayer plate C) was conducted approximately 1 month after the previous assays. However, as the comparison is between cell culture template and serially diluted template from the same oocysts this was not deemed to be a major limitation. Monolayer inoculation plate A was run with stock oocyst dilution series A and monolayer inoculation plate B with oocyst dilution series B. Each DNA template was run in duplicate on each plate. Cycling conditions were as follows: 95 °C for 2 min, followed by 44 cycles (95 °C for 10 s, 58 °C for 10 s, 72 °C for 20 s) on a C1000 Touch thermal cycler (BioRad). BioRad CFX Manager Software [38] was used to analyse the amplification curves. Quantification cycle was set at the beginning of the log-linear phase of the amplification plot where the variation of Cq-values between technical repeats were minimal. Non-template controls (NTC) were included on each qPCR plate with Cq ≥ 40 considered negative [39].

### Flow cytometry

A 10-fold dilution of oocysts (5 × 10^4^–0.5; 5 replicates for each suspension) in maintenance medium (100 μl) was seeded onto a 96-well plate with a multi-channel pipette without cells and inter-well variation assessed by flow cytometry with the NovoCyte NovoSampler (Acea Biosciences Inc, Santa Clara, CA, USA), sample volume 60 μl, threshold set to Forward scatter -H (FS-H) < 300. Output was analysed with NovoExpress software and samples were gated based on blank control background readings to only include oocyst counts.

### Qubit™ DNA quantification

Qubit™ DNA quantification was performed with Qubit™ 1× double stranded DNA (dsDNA) HS Assay Kit (Thermo Fisher Scientific, Waltham, MA, USA). Ten microlitres of DNA template from each biological repeat from cell inoculation plate C (5 × 10^4^–500) and equivalent concentrations of freely suspended oocysts were added to 190 µl Qubit™ 1× dsDNA HS Working Solution. Samples were vortexed (5 s) and centrifuged (5 s) prior to being analysed on an Invitrogen Qubit 4 fluorometer (Thermo Fisher Scientific). DNA content was recorded as ng/µl calibrated with Qubit™ 1× dsDNA HS Standard #1 and Qubit™ 1× dsDNA HS Standard #2 included in the kit.

### Statistics

Differences between Cq-values on the same plate were assessed *via* linear regression. Differences were considered significant if *P* ≤ 0.05. All data was analysed in R [40]. Standard curves were produced in BIORAD CFX Manager v3.1 [38].

## Results

### Oocyst monolayer inoculations: Plates (i) and (ii)

A substantial loss in cell viability was observed in all wells inoculated with 10^5^ oocysts for inoculation plate A (50 ± 11.4% compared with blank wells). For plate B, 3/5 wells exhibited OD_450_ values > 75% (82.4 ± 6.5%), a threshold previously considered not to constitute toxicity in pharmaceutical screenings [11]. The remaining two wells had OD_450_ values 66.9 and 9.1% when compared to the blank controls. Laboratory error resulted in marked pipette damage on the cell monolayer to the latter and was thus excluded from all subsequent analyses.

Average Cq-values across biological repeats for Plate (i) were 28.76 ± 0.81, 29.69 ± 0.38, 33.45 ± 1.09, 36.5 ± 0.66, 41.28 ± 2.54 and 42.66 ± 1.19 for oocyst inoculates of 10^5^ to 1/well, respectively (*R*^2^ = 0.899). Two technical repeats (well 4, 10^5^ oocysts, and well 5, 1 oocyst) were clear outliers due to experimental error and were removed from the analysis. For Plate (ii) Cq values were: 28.91 ± 0.85, 30.43 ± 0.88, 34.73 ± 0.74 and 37.39 ± 0.6 for oocyst inoculates 10^5^ to 10^2^ (*R*^2^ = 0.901). Average Cq-values for one oocyst across biological repeats was 43.27 ± 1.26. For inoculates of 10 oocysts, amplification of DNA was observed in only one well for both technical repeats, Cq of 41.06 ± 0.77 for Plate (ii). Inactivated oocysts inoculated at 10^5^/well indicated Cq-values across biological repeats of 36.34 ± 0.56 for Plate (i) and 37.39 ± 0.72 for Plate (ii). Cq-values for inactivated oocysts were not significantly different from those obtained from 100 stock oocyst inoculate (*P* = 0.7 and 0.99 for Plate (i) and (ii), respectively) but were significantly different for all other concentrations (Additional file 1: Table S1, Additional file 2: Table S2).

### Stock oocyst dilutions: Plate (iii)

For 10-fold dilutions of pure stock oocysts, average Cq-values across two technical repeats were 29.45 ± 0.42, 31.34 ± 31.34 and 34.61 ± 0.28 for 10^6^, 10^5^ and 10^4^ oocysts, respectively for serial dilution A (*R*^2^ = 0.971). For 10^3^ oocysts, one technical repeat failed to amplify any DNA with a Cq-value of 38 for the other. For serial dilution B, Cq-values were 28.74 ± 0.42, 30.29 ± 0.16, 33.86 ± 0.22 and 37.84 ± 0.75 for 10^6^, 10^5^, 10^4^ and 10^3^ oocysts, respectively. No signal was detected for DNA from 10^2^, 10^1^ and 1 for either dilution series (*R*^*2*^ = 0.968). No signal was observed for MilliQ water negative controls, as expected (Additional file 3: Table S3).

### Stock oocyst dilutions and DNA template dilutions: Plate (iv)

Ten-fold dilutions of stock oocysts yielded Cq-values of 28.9 ± 0.8, 31.09 ± 0.25, 34.24 ± 0.03, 38.17 ± 0.75 and 43.34 ± 0.5 for 10^6^, 10^5^, 10^4^, 10^3^ and 10^2^ oocysts, respectively in serial dilution A (*R*^2^ = 0.967) and 27.62 ± 0.67, 30.35 ± 0.15, 33.64 ± 0.24, 37.95 ± 0.56 and 40.82 ± 0.34 for 10^6^, 10^5^, 10^4^, 10^3^ and 10^2^ in serial dilution B (*R*^2^ = 0.956). Similar reductions in Cq-values were observed for corresponding dilutions of these templates: 31.04 ± 0.35, 34.09 ± 0.53, 38.11 (one technical repeat failed to amplify DNA) and 40.82 ± 0.34 for ×10, ×10^2^, ×10^3^ and ×10^4^ dilutions, dilution A (*R*^2^ = 0.987). DNA template dilutions of serial dilution B gave Cq-values of 30.52 ± 0.17, 35.11 ± 0.04, 38.19 ± 0.08 and 41.37 (one technical repeat failed to amplify DNA) for ×10, ×10^2^, ×10^3^ and ×10^4^ dilutions (*R*^2^ = 0.951). No DNA was amplified at the ×10^5^ or ×10^6^ dilutions. No signal was observed for the MilliQ water negative control (Additional file 4: Table S4).

### Oocyst monolayer inoculations, stock oocyst dilutions and DNA template dilutions: Plates (v) and (vi)

**Fig. 2:**
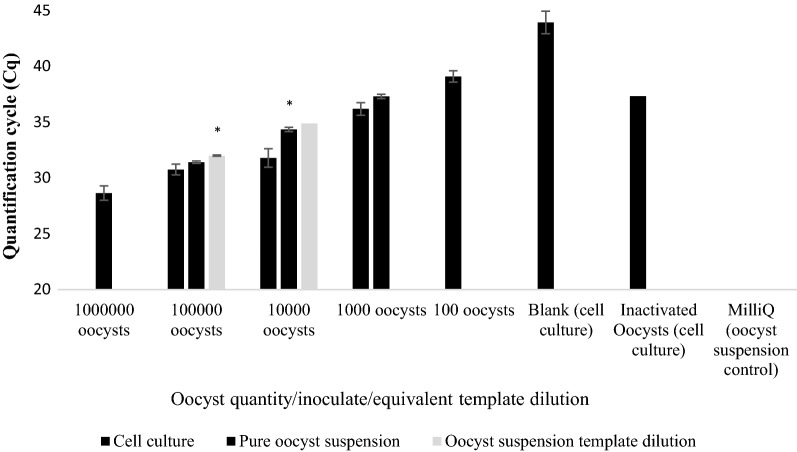
Average differences in Cq-values for quantitative PCR Plate (v) between *Cryptosporidium parvum* oocysts inoculated onto HCT-8 monolayer plate A, pure oocyst serial dilution A and equivalent template dilution A. Error bars represent the standard deviation of the mean (SD) from 3 biological repeats for cell culture data and SD of two technical repeats for the pure oocyst serial dilution and equivalent template dilution data. Asterisks indicate significantly increased Cq signal (*P* < 0.05) compared to cell culture derived values

**Fig. 3:**
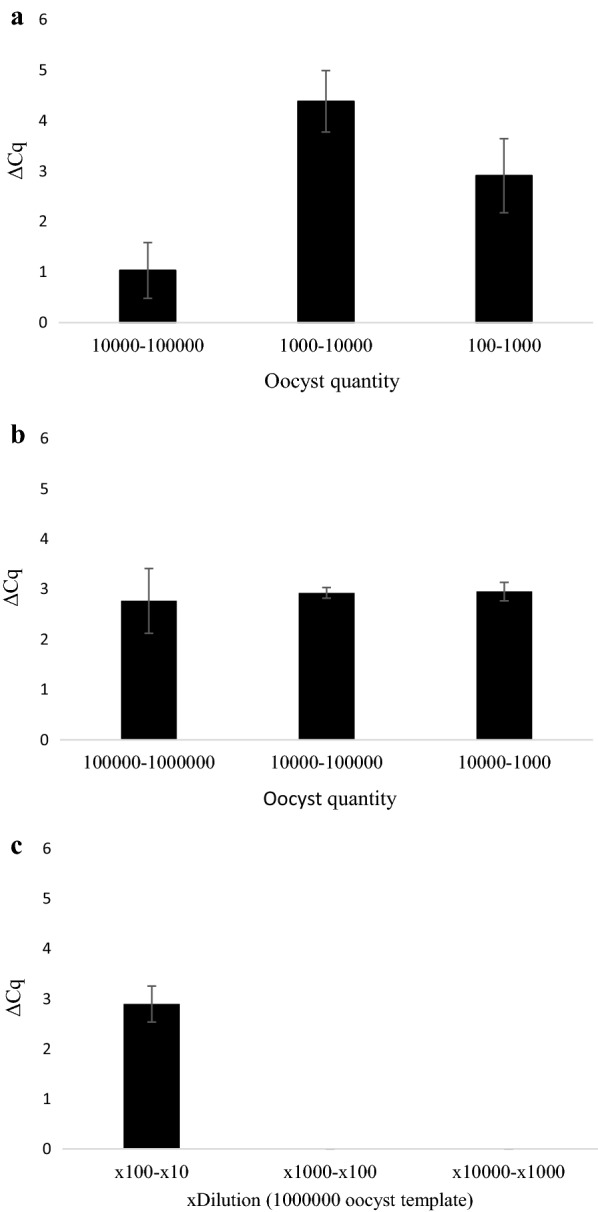
Change in Cq-values (ΔCq) (Plate (v)) between serial dilutions of *Cryptosporidium parvum* oocysts in inoculated into cell-culture (**a**), pure oocyst suspensions (**b**) and 10^6^ oocysts DNA template (**c**). ΔCq was calculated by subtracting the Cq-value of the lower concentration from the immediate higher concentration

**Fig. 4:**
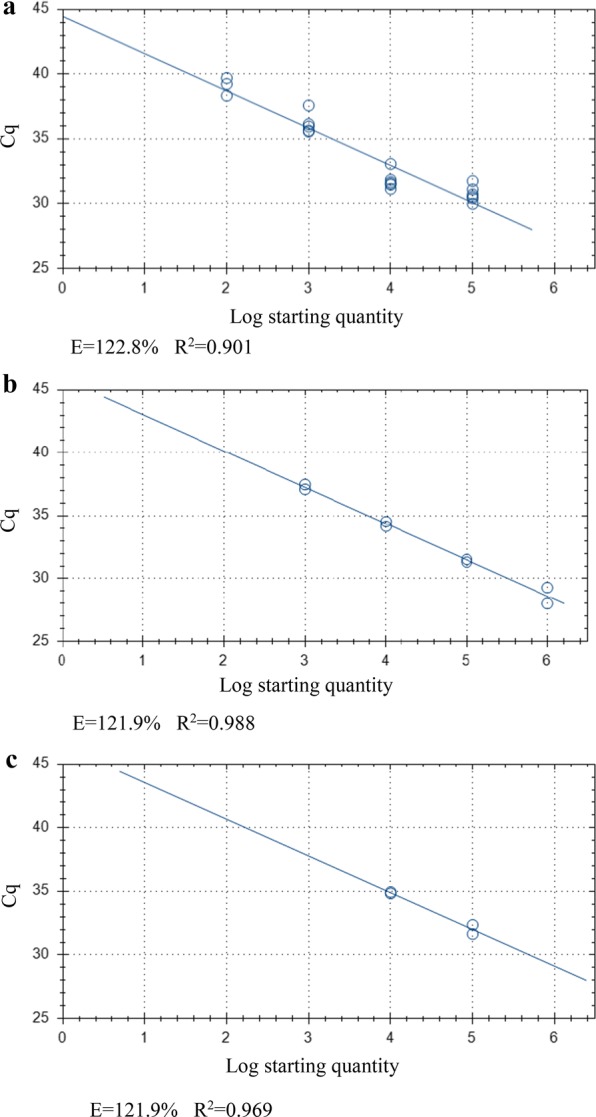
Standard curve generated for Plate (v) from *Cryptosporidium parvum* oocysts inoculated onto HCT-8 cell monolayer plate A (10^5^–10^2^) (**a**), pure oocyst serial dilution A (10^6^–10^2^) (**b**) and equivalent template dilution A (**c**). *Abbreviations*: E, qPCR amplification efficiency; *R*^2^, coefficient of variation

Average Cq-values across biological repeats were 30.76 ± 0.5, 31.79 ± 0.48 and 36.18 ± 0.85 for 10^5^, 10^4^ and 10^3^ oocyst inoculates, respectively for Plate (v). With Cq-values from all biological and technical repeats pooled, change in Cq (ΔCq) was 1.03 (10^5^–10^4^, *P* = 0.017), 4.33 (10^4^–10^3^, *P* < 0.0001) and 4.91 (10^3^–10^2^, *P* = 0.002) (Table 1, Figs. 2, 3a), with a linearity of *R*^2^ = 0.901 and amplification efficiency of 122.8% (Fig. 4a), but 100 oocysts per well inoculates failed to generate a positive signal across both technical repeats for any well. The average ΔCq-value between concentrations was 2.78 ± 1.37. The blank controls produced signal for two technical repeats in separate wells (43.93 ± 0.51) with one reaction containing inactivated oocysts producing a signal (37.33 ± 1.00) in both repeats. The highest four 10-fold stock oocyst dilutions from 10^6^ produced Cq-values of 28.65 ± 0.65, 31.42 ± 0.11, 34.35 ± 0.19 and 37.3 ± 0.2, with 100 oocysts remaining negative. Average ΔCq-values for 10-fold dilutions were 2.89 ± 0.08 (10^6^–10^5^, *P* = 0.051), 2.93 (10^5^–10^4^, *P =* 0.005), 2.96 (10^4^–10^3^, *P* = 0.008) (*R*^2^ = 0.988, amplification efficiency of 121.9%; Table 2, Figs. 2, 3b, 4b). A similar association was found with DNA template dilutions, 32 and 34.90, for the ×10 and ×10^2^ dilutions, respectively, with an average ΔCq of 3.45 ± 0.175 (*R*^2^ = 0.969, amplification efficiency of 121.9%; Table 3, Figs. 2, 3c, 4c), with no significant difference between these and the stock oocyst dilutions in Plate (v). However, the ×10^3^ and ×10^4^ dilutions failed to produce signal. Significantly lower Cq-values were found for the same oocyst numbers grown in cell culture for 10^5^ in comparison to the equivalent template dilution (*P =* 0.028) but not the pure oocyst suspension (*P* = 0.188). For 10^4^ suspensions and the diluted template DNA equivalent, cell cultured oocysts had significantly lower Cq-values than both the stock dilution and template DNA dilution (*P =* 0.009 and 0.003 for stock and template dilutions, respectively). For 10^3^ oocysts, no difference was found between cell cultured oocysts and pure oocyst dilutions (*P* = 0.132) and no signal was observed in the equivalent diluted template. No signal was observed for 10^2^ stock oocysts nor the respective diluted and the three technical repeats failed to amplify in the cell culture wells. No significant differences were observed between stock oocyst dilutions and the templates at the highest two template concentrations (i.e. the two concentrations where DNA signal was produced across all three DNA standard types).

**Table 1:** Cq values of quantitative PCR Plate (v)

Oocyst inoculate		Well 1		Well 2		Well 3	
		TR1	TR2	TR1	TR2	TR1	TR2
100,000		31.75	31.11	30.72	30.55	30.42	30
Mean ± SD	TR	31.43 ± 0.32		30.64 ± 0.09		30.21 ± 0.21	
	BR	30.76 ± 0.50					
10,000		31.48	31.66	31.15	31.54	31.85	37.07
Mean ± SD	TR	31.57 ± 0.09		31.35 ± 0.19		32.46 ± 0.19	
	BR	31.79 ± 0.48					
1000		na	37.59	35.96	35.64	36.14	35.57
Mean ± SD	TR	na		35.8 ± 0.16		35.86 ± 0.29	
	BR	36.18 ± 0.85					
100		39.24	na	38.34	na	na	39.7
Mean ± SD	TR	na		na		na	
	BR	na					
Blank wells		na	43.42	na	na	44.44	na
Mean ± SD	TR	na					
	BR	43.92 ± 0.51					
Inactivated oocysts		na	na	38.33	36.32	na	na
Mean ± SD	TR	na		37.33 ± 1.00		na	na
	BR	37.33 ± 1.00					

*Notes*: *Cryptosporidium parvum* oocysts inoculated onto HCT-8 cell monolayer plate A. Two technical repeats for each well (biological repeat). Baseline threshold 11.89 RFU

*Abbreviations*: TR, technical repeat; BR, biological repeat; SD, standard deviation; na, no DNA amplified

**Table 2:** Cq values of quantitative PCR Plate (v)

Oocyst quantity	TR1	TR2
1,000,000	29.29	28
Mean TR ± SD	28.65 ± 0.65	
100,000	31.52	31.31
Mean TR ± SD	31.42 ± 0.11	
10,000	34.16	34.53
Mean TR ± SD	34.35 ± 0.19	
1000	37.11	37.49
Mean TR ± SD	37.3 ± 0.20	
100	na	na
Mean TR ± SD	na	
MQ	na	na
Mean TR ± SD	na	

*Notes:* Pure oocyst serial dilution A. Baseline threshold 11.89 RFU

*Abbreviations:* TR, technical repeat; SD, standard deviation; na, no DNA amplification

**Table 3:** Cq values of quantitative PCR Plate (v)

Template dilution	TR1	TR2
×10	32.36	31.64
Mean TR ± SD	32 ± 0.36	
×100	34.84	34.95
Mean TR ± SD	34.9 ± 0.06	
×1000	na	na
Mean TR ± SD	na	
×10,000	na	na
Mean TR ± SD	na	

*Notes*: DNA template dilution of 10^6^ oocyst suspension A. Baseline threshold 11.89 RFU

*Abbreviations:* TR, technical repeat; SD, standard deviation; na, no DNA amplification

**Fig. 5:**
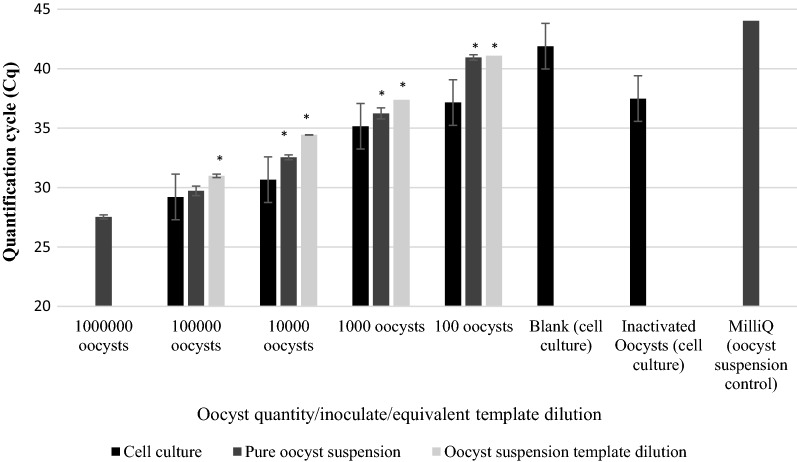
Average differences in Cq-values for quantitative PCR Plate (v) between *Cryptosporidium parvum* oocysts inoculated onto HCT-8 monolayer plate B, pure oocyst serial dilution B and equivalent template dilution B. Error bars represent the standard deviation of the mean (SD) from 3 biological repeats for cell culture data and SD of two technical repeats of the pure oocyst serial dilution and equivalent template dilution data. Asterisks indicate significantly increased Cq signal (*P* < 0.05) compared to cell culture-derived values

**Fig. 6:**
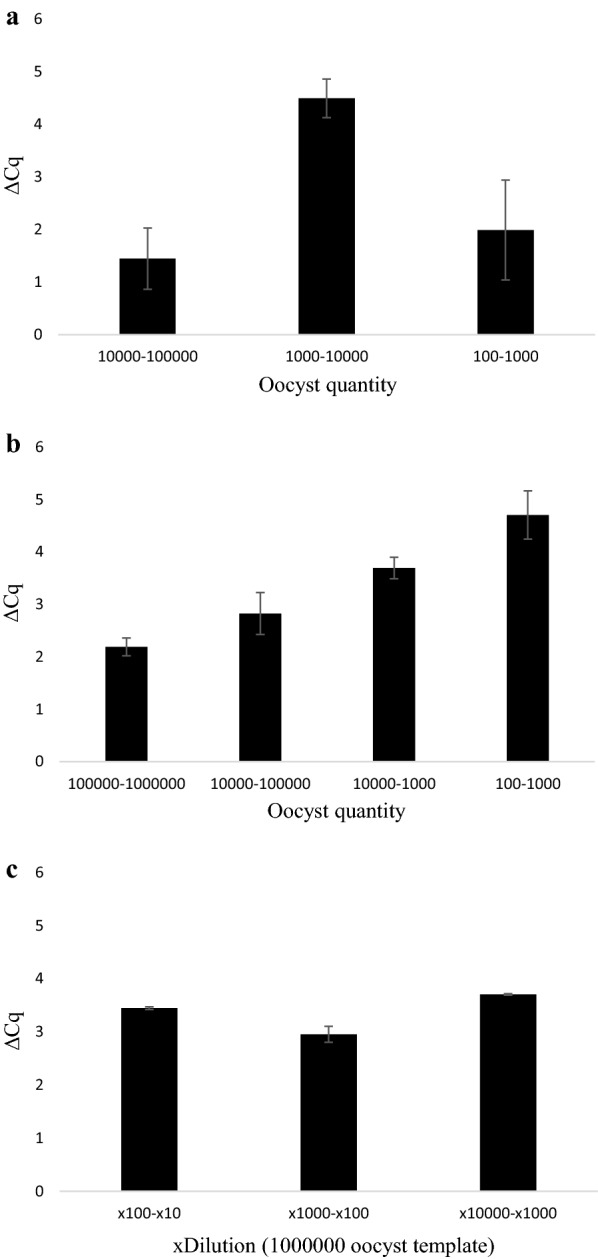
Change in Cq-values (ΔCq) (Plate (vi)) between serial dilutions of *Cryptosporidium parvum* oocysts inoculated into cell culture (**a**), in pure oocyst suspensions (**b**) and 10^6^ oocysts DNA template (**c**). ΔCq was calculated by subtracting the Cq-value of the lower concentration from the immediate higher concentration

**Fig. 7:**
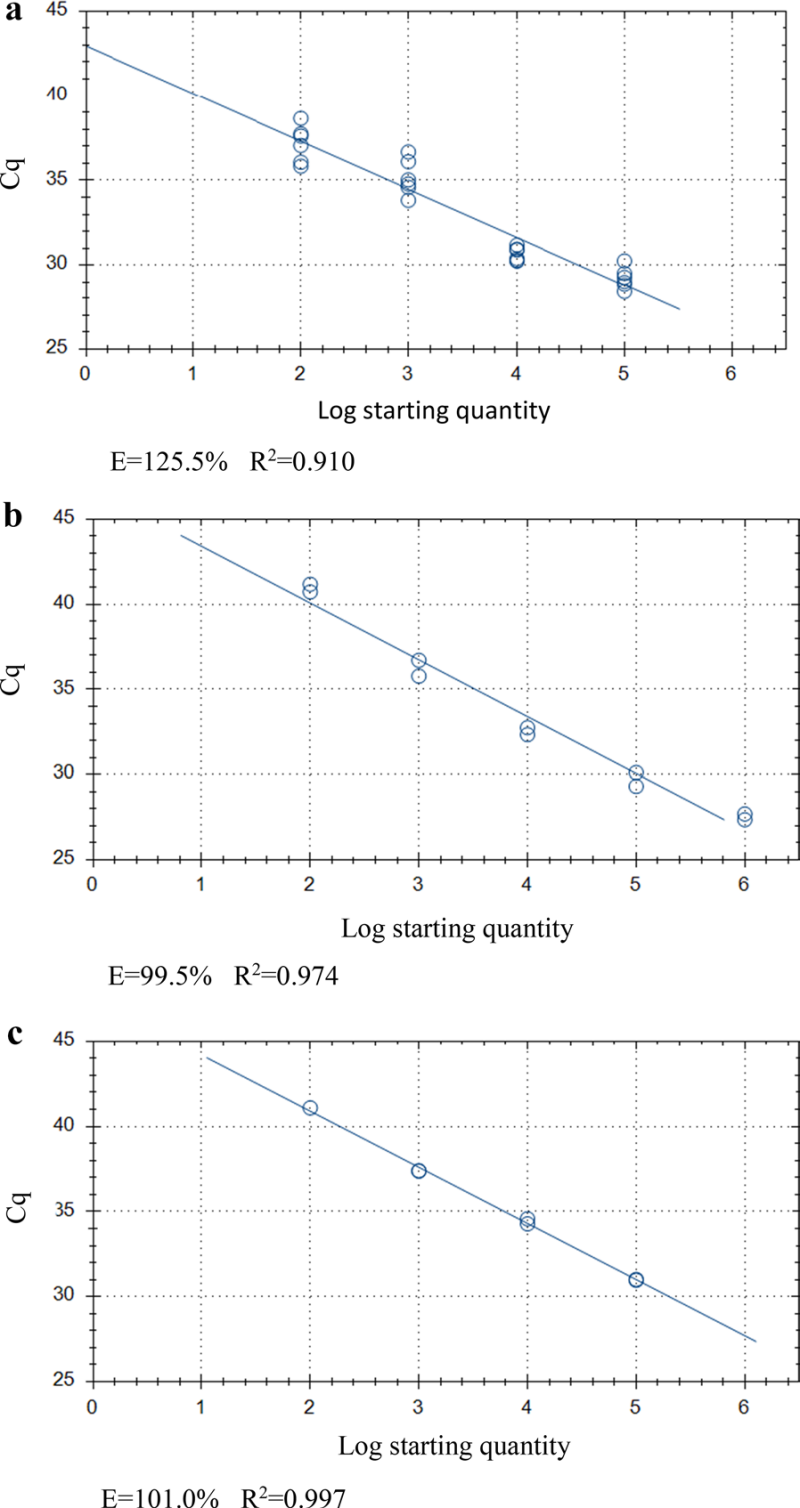
Standard curve generated for Plate (vi) from *Cryptosporidium parvum* oocysts inoculated onto HCT-8 cell monolayer plate B (10^5^–10^2^) (**a**), pure oocyst serial dilution B (10^6^–10^2^) (**b**) and equivalent template dilution B (**c**). *Abbreviations*: E, qPCR amplification efficiency; *R*^2^, coefficient of variation

For Plate (vi), the average Cq-values across biological repeats in cell cultured oocysts were 29.21 ± 0.50, 30.66 ± 0.30, 35.16 ± 0.90 and 37.15 ± 0.86 for 10^5^–10^2^ oocyst inoculates. With Cq-values from all biological and technical repeats pooled, ΔCq-values were 1.44 (1 × 10^5^–1 × 10^4^, *P* < 0.001), 4.5 (10^4^–10^3^, *P* < 0.0001) and 2 (10^3^–10^2^, *P* = 0.008). The average ΔCq was 2.65 ± 1.33 (*R*^2^ = 0.910, amplification efficiency of 125.5%; Table 4, Figs. 5, 6a, 7a). For inactivated oocysts, the average Cq-value across biological repeats was 37.49 ± 0.35. Serial stock oocyst dilution values were 27.53 ± 0.4, 29.72 ± 0.21, 36.24 ± 0.46 and 40.95 ± 0.23 for 10^6^–10^2^ oocyst inoculates. ΔCq-values were 2.19 (10^6^–10^5^, *P* = 0.04), 2.85 (10^5^–10^4^, *P* = 0.024), 3.7 (10^4^–10^3^, *P* = 0.018) and 4.71 (10^3^–10^2^, *P* = 0.01). The average ΔCq was 2.9 ± 0.62 (*R*^2^ = 0.974, amplification efficiency of 99.5%; Table 5, Figs. 5, 6, 7b). For the template DNA dilutions the average Cq-values were 30.99 ± 0.03, 34.43 ± 0.15 and 37.39 ± 0.02 (×10^1^, ×10^1^ and ×10^2^ dilutions, respectively; only one technical repeat was amplified for the ×10^3^ dilution, Cq = 41.1). ΔCq-values for the template DNA dilutions were 3.45 and 2.94 (×10–×10^2^, *P* = 0.002; and ×10^2^–×10^3^, *P* = 0.0026 dilutions, respectively). The average ΔCq was 3.37 ± 0.31 (*R*^2^ = 0.997, amplification efficiency of 101%; Table 6, Figs. 5, 6, 7c). One hundred thousand oocysts in cell culture did not produce significantly lower Cq-values than diluted stock oocysts, but they did for the equivalent DNA template (*P =* 0.007); for 10^4^, both stock dilution and equivalent template dilutions having significantly higher Cq-values than cell culture (*P* = 0.0004 and < 0.0001, stock and template dilutions, respectively) and for 10^3^ (*P =* 0.003 and 0.001, stock and DNA template dilutions, respectively). One hundred oocysts in cell culture produced significantly lower Cq-values compared to stock oocyst dilutions (*P* = 0.003) and the equivalent template dilutions (*P* = 0.001). No significant differences were found between the stock oocyst dilutions and their equivalent DNA template dilution with the exception of 10^4^, where the Cq-value of the template was significantly higher than that of the stock dilution (*P* =. 0.0013). Inactivated oocyst Cq-values were not significantly different to values obtained from 10^2^ oocyst inoculate in cell culture (*P* = 0.464).

**Table 4:** Cq values of Quantitative PCR Plate (vi)

Oocyst inoculate		Well 1		Well 2		Well 3	
		TR1	TR2	TR1	TR2	TR1	TR2
100,000		30.25	29.52	28.29	28.83	28.4	28.98
Mean ± SD	TR	28.89 ± 0.37		29.06 ± 0.23		28.63 ± 0.29	
	BR	29.21 ± 0.50					
10,000		30.24	30.90	30.29	30.39	30.95	31.19
Mean ± SD	TR	30.57 ± 0.33		30.34 ± 0.05		31.07 ± 0.12	
	BR	30.66 ± 0.30					
1000		36.66	36.09	34.78	33.82	34.58	35.02
Mean ± SD	TR	36.38 ± 0.29		34.3 ± 0.48		34.8 ± 0.22	
	BR	35.16 ± 0.90					
100		35.83	36.06	37.6	37.73	37.04	38.64
Mean ± SD	TR	35.95 ± 0.12		37.67 ± 0.07		37.84 ± 0.80	
	BR	37.15 ± 0.86					
Blank wells		na	42.80	44	41.28	39.49	na
Mean ± SD	TR	na		42.64 ± 1.36		na	
	BR	41.9 ± 1.69					
Inactivated oocysts		37.23	37.46	37.7	38.23	36.63	37.67
Mean ± SD	TR	37.35 ± 0.12		37.97 ± 0.27		37.15 ± 0.52	
	BR	37.49 ± 0.35					

*Notes*: *C. parvum* oocysts inoculated onto HCT-8 cell monolayer plate B. Two technical repeats for each well (biological repeat). Baseline threshold 19.88 RFU

*Abbreviations*: TR, technical repeat; BR, biological repeat; SD, standard deviation; na, no DNA amplification

**Table 5:** Cq values of quantitative PCR Plate (vi)

Oocyst quantity	TR1	TR2
1,000,000	27.70	27.36
Mean TR ± SD	27.53 ± 0.17	
100,000	30.12	29.32
Mean TR ± SD	29.72 ± 0.40	
10,000	32.75	32.34
Mean TR ± SD	32.55 ± 0.21	
1000	36.70	35.78
Mean TR ± SD	36.24 ± 0.46	
100	41.17	40.72
Mean TR ± SD	40.95 ± 0.23	
MQ	na	na
Mean TR ± SD	na	

*Notes*: Pure oocyst serial dilution B. Baseline threshold 19.88 RFU

*Abbreviations*: TR, technical repeat; SD, standard deviation; na, no DNA amplification

**Table 6:** Cq values of quantitative PCR Plate (vi)

Template dilution	TR1	TR2
×10	31.07	30.96
Mean TR ± SD	30.99 ± 0.03	
×100	34.28	34.58
Mean TR ± SD	34.43 ± 0.20	
×1000	37.37	37.4
Mean TR ± SD	37.39 ± 0.02	
×10,000	na	41.49
Mean TR ± SD	na	

*Notes*: DNA template dilution of 10^6^ oocyst suspension B. Baseline threshold 19.88 RFU

*Abbreviations*: TR, technical repeat; SD, standard deviation; na, no DNA amplification

### Oocyst monolayer inoculations and DNA template dilutions: Plate (vii)

**Fig. 8:**
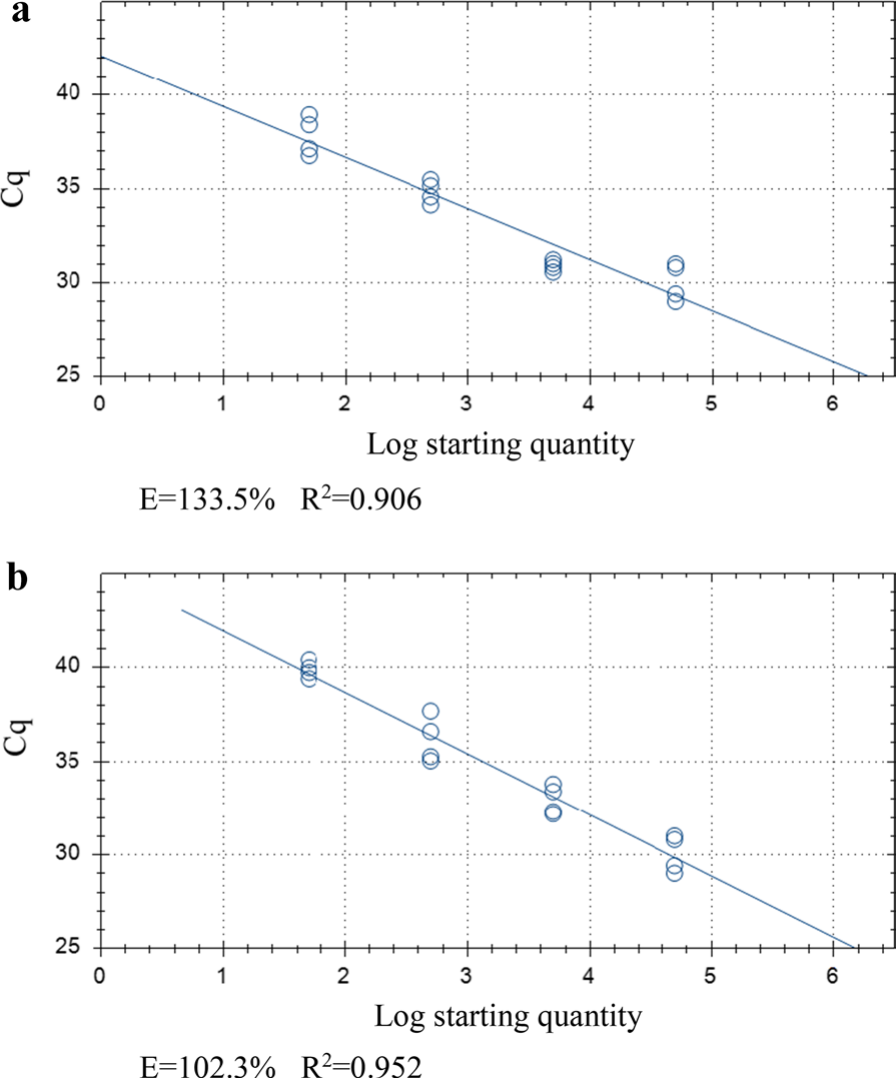
Standard curve generated for Plate (vii) from *Cryptosporidium parvum* oocysts inoculated onto HCT-8 cell monolayer plate C (5 × 10^4^–5 × 10^1^) (**a**) and cell culture serial dilution of C and D (×10–1000) (**b**). *Abbreviations*: E, qPCR amplification efficiency; *R*^2^, coefficient of variation

**Fig. 9:**
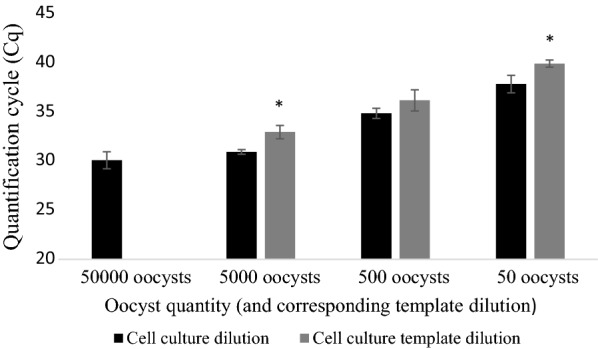
Average Cq-values of quantitative PCR for Plate (vii). *Cryptosporidium parvum* oocysts inoculated onto HCT-8 monolayer plate C (5 × 10^4^–50) and 10-fold dilutions of 5 × 10^4^ (×10, ×100 and ×1000). Error bars represent the standard deviation of the mean (SD) from 2 biological repeats for cell culture data and SD from two technical repeats of each dilution for the template dilution data. Asterisks indicate significantly increased Cq signal (*P* < 0.05) compared to cell culture derived values

Cq-values across biological repeats in cell cultured oocysts were 30.07 ± 0.9, 30.91 ± 0.24, 34.84 ± 0.52 and 37.81 ± 0.89 for 5 × 10^4^–50 oocyst inoculates (*R*^2^ = 9.06, amplification efficiency of 133.5%; Fig. 8a). For the 10-fold dilution of the 5 × 10^4^ cell culture standards, Cq-values (averaged across both dilution series) were 32.93 ± 0.68, 36.15 ± 1.07 and 39.89 ± 0.36 for ×10–×1000 cell cultured DNA template dilutions, respectively (*R*^2^ = 0.952, amplification efficiency of 102.3%; Fig. 8b). Significantly lower Cq-values were obtained in cell cultured oocysts at 5 × 10^3^ and 50 compared to the equivalent diluted template (*P =* 0.003 and 0.01, respectively) (Fig. 9).

### Flow cytometry

Flow cytometric analysis revealed relatively low variation for oocyst inoculates seeded at high concentration suspensions (± 7.0% and 7.2% for 5 × 10^4^ and 5 × 10^3^, respectively) but at lower seeding densities variation increased substantially (± 31.63%, 22.2%, 16.73% and 45.7% for 5 × 10^2^, 5 × 10^1^, 5 and 0.5 oocysts, respectively).

### Qubit™ DNA quantification

The average DNA concentration for cell cultured samples was 6.08 ± 1.12, 9.1 ± 0.78 and 10.08 ± 0.56 ng/µl from 5 × 10^4^–500 oocyst inoculates with 5 × 10^4^ freely suspended oocysts averaging 0.045 ± 0.005 ng/µl. DNA content was too low in all lower concentrations of freely suspended oocysts for signal detection.

## Discussion

This study clearly demonstrates important differences in the results generated between cell-culture incubated oocysts, pure oocyst dilutions and their respective DNA template equivalents when used as standards for qPCR. Their use as standards in experiments could influence the results if precise data are needed, as the results demonstrate important differences in linearity and ΔCq between the methods. To the best of our knowledge, this is the first study to compare different protocols for preparing qPCR standards for *C. parvum* and their comparative results when calculating parasite content.

In plates (v) and (vi), cell culture Cq-values in many samples were significantly lower than values observed for stock oocyst dilutions and the equivalent template dilutions although this was not found uniformly across all oocyst concentrations. For 10^5^ both plates produced significantly lower Cq-values (i.e. indicating that more parasite DNA was present) in cell culture, compared to the diluted templates, but not for pure oocyst suspensions. For Plate (vi), all subsequent lower concentrations produced significantly lower Cq-values in cell culture compared to the stock oocyst and template dilutions, but for Plate (v), this was only the case for 10^4^ oocysts. No significant difference in Cq-values between stock oocyst suspensions and cell culture were documented at 10^3^ oocysts. The lack of significantly higher quantities of DNA in the cell culture samples at 10^5^ oocysts may be attributable to the loss of cell viability in these wells although this is less likely to be a factor for Plate (vi). Cell stress as a result of a high oocyst inoculates is the likely cause of this loss of viability and a prospective study should assess any potential differences at lower oocyst inoculates. No DNA was amplified in any plates for stock oocyst dilutions and equivalent templates when there were < 100 oocysts.

The higher number of DNA copies obtained in cell culture inoculations compared with equivalent pure oocyst suspensions (or diluted DNA template of pure oocysts) is indicative of parasite growth. Once sporozoites have invaded the host cell, they undergo asexual and sexual multiplication and within 24 hours have produced meronts I [31] each containing 6–8 merozoites [13]. As each oocyst contains 4 sporozoites that will each individually perform asexual multiplication with subsequent later stages in the life-cycle reproducing sexually and although it is unclear which stages are specifically being amplified in cell culture [31], more DNA will be produced through growth.

For plates (v) and (vi) the standard deviation (SD) between technical repeats was < 0.5 for all samples indicating high reproducibility [41]. However, these same templates, when analysed on plates (i), (ii), (iii) and (iv) exhibited variation > 0.5 for some samples highlighting the influence of pipetting error during reaction mix or DNA template application. Supporting this, detection limits varied between plates especially for stock oocyst dilutions and their equivalent templates. Plate (iii) exhibited no detection of 100 stock oocysts and Plate (v) exhibited neither detection of 100 oocysts nor the ×10^4^ dilution despite these exact same templates amplifying DNA in Plate (iv). One hundred oocysts in cell culture on Plate (v) did not yield any Cq signal in one technical repeat of each well despite amplification being observed for the same templates in Plate (i) and Plate (ii), although one technical repeat for Plate (ii) failed to amplify for the ×10^4^ template dilution in Plate (ii). Laboratory error during pipetting the template onto the qPCR plate may explain the lack of detection in instances where signal is not produced in one technical repeat for each well (biological repeat). This could include insufficient or no template being placed in the qPCR plate well. In order to mitigate this in the future it would be optimal to increase the quantity of DNA template in the reaction. As it was imperative that the same templates were used multiple times to insure robust comparison between methods, we were only able to use 0.5 µl in the present study. This may also explain the significantly higher Cq-value in the 10^4^ equivalent template dilution on Plate (vi) when all other concentrations were not statistically different. Although Cq-values varied between plates, overall, there was a consensus between the same templates analysed on different plates.

For subsequent studies, variation in seeding densities of oocysts and excessive variation between Cq-values of technical repeats, automated techniques should be considered. These have the potential to reduce variation due to experimental error caused by human operators. However, these are expensive and therefore unavailable in many laboratories. If automated techniques are unavailable, studies should consider increasing the number of repeats, both biological and technical, in order to mitigate the problems caused by high variation.

Mean Cq-values for inactivated oocysts were not significantly different from that of the 100 oocyst inoculates in cell culture in all four independent plates in which they were included, i.e. (i), (ii), (iii) and (iv), with all other concentrations exhibiting significantly higher or lower Cq-values. This differs from the results obtained by Keegan et al. [23] where serially diluted inactivated oocysts (10^5^–1) were not detected in the qPCR assay, indicating the complete removal of unexcysted oocysts and oocyst debris from the monolayer. Washing steps were similar with 3× PBS washes per well in both studies although Keegan et al. [23] used 24-well plates and washed with 500 μl PBS as opposed to 96-well plates with 100 μl PBS used in the present study (although PBS/well surface area was approximately the same). It is highly unlikely that the signal produced in the inactivated oocysts wells was due to contamination as blank wells that produced signal averaged Cq of 41.8 ± 2 across biological repeats opposed to 36.34 ± 0.56 for inactivated oocysts representing a 5.4 difference in Cq. Although direct comparison between plates is not appropriate, it should be noted that 100 stock oocyst suspensions yielded either no signal, or Cq-values > 40 which is indicative of oocyst invasion in the inactivated oocyst wells. If so, as 10^5^ inactivated oocysts were added to each well, this represents 99.9% oocyst inactivation and incubation for 10 min at 80 °C [23] may be preferable to 70 °C for 30 min.

Dilution of the 10^6^ stock oocyst template did not yield significantly different Cq-values compared to stock oocyst dilutions except for the lower Cq-values in 10^4^ stock oocysts and its equivalent diluted template in Plate (vi). Detection limits of stock dilutions of suspended oocysts were higher than previously reported with similar qPCR designs yielding sensitivity down to one oocyst or lower [42, 43]. In addition to the reduced template quantity in the qPCR reaction described above, other factors, such as varying compositions of PCR master mix, primers, probes, water, oocyst strain and age may also account for this. Primer design and target regions appear to be particularly important. In one study comparing 16 different primer designs and target regions, only 7 enabled detection of *C. parvum* oocysts at numbers < 10^4^. Furthermore, as oocyst numbers in a sample decreased, the percentage of positive signals produced for a particular quantity of oocysts also reduced for certain primers [44]. It is important to note that all these factors are relative to the specific assay and as all conditions were uniform for all comparisons in this study, we feel confident that reduced detection limits in these assays do not compromise the findings of our study. Prospective studies should focus on a better understanding of the differences between these different standard types at lower concentrations.

Linearity values were also closer to 1 for both the stock oocyst dilutions and their equivalent templates than they were for cell culture derived templates. These linearity differences between the three DNA templates were maintained across all plates, further supporting the reproducibility of the assay. This is likely a consequence of the inherent variation encountered in *C. parvum* cell culture [9], which is supported by the increase in seeding variation revealed by flow cytometry in our study, with inter-well variation increasing at decreasing inoculate concentrations. As such, even for studies employing cell culture, stock oocysts or diluted template DNA may be desirable. Alternatively, considering the similarity of the template dilutions, serial dilutions of cell culture template DNA may provide the ideal standards for studies assessing *Cryptosporidium* growth.

As shown in Figs. 3 and 6, ΔCq-values from cell culture derived oocysts were less uniform across inoculation ranges than their pure oocyst and oocyst DNA template dilution equivalents. Lower ΔCq was observed in cell culture between 10^5^ and 10^4^ oocysts compared to 10^4^ and the subsequent dilutions (10^3^–10^2^) in plates (i), (ii), (v) and (vi). The reduced viability of the cells inoculated with 10^5^ oocysts may in part be responsible for this finding; however, this was also observed in Plate (iv), where cell viability values of the 10^5^ samples analysed all had OD_450_ values within 75% of the blank control wells. This could suggest that possible inhibitors are present in the reaction mix; however, it is unlikely that the inhibitors originate from the host cells as these were seeded at the same density for all samples. The reduced ΔCq at the highest concentrations of stock oocysts and diluted template DNA was not as marked as in cell culture, although was still lower than differences between lower concentrations. This was initially thought to be a result of human DNA in the cell culture standards exceeding the binding capacity of the DNA extraction column resulting in < 100% *C. parvum* DNA present in the extracted template. However, DNA content in cell culture standards from plate C did not exceed 10.64 ± 0.56 ng/µl (although it exceeded DNA content in equivalent quantities of freely suspended oocysts). The DNeasy® Blood & Tissue Kit (Qiagen) is capable of extracting, on average, 30 µg DNA from lysed 25 µg tissue sample [45], greatly in excess of DNA quantities present in the cell culture standards. DNA saturation in the reaction mix is a further potential reason for this reduced ΔCq at higher concentrations, however this is challenged by 10^6^ stock oocysts producing significantly lower Cq-values than 10^5^ oocysts in cell culture.

Cq-values from serially diluted oocysts did not differ significantly from the DNA template dilutions of 10^6^ oocysts and if inhibitors were present, lower Cq-values would be expected in the diluted template [11]. However, amplification efficiency values in cell cultured oocysts were > 100% in all cell cultured standards in plates (v), (vi) and (vii) (Figs. 4a, 7a, 8a) with freely suspended oocysts and the DNA template dilutions of freely suspended oocysts and cell culture standards yielding amplification efficiency values closer to 100% on plates (vi) and (vii). Amplification efficiencies > 100% are indicative of polymerase inhibition [44], so it seems likely that this is, at least in part, responsible for the reduced linearity observed in the cell culture standards. Substances present in the HCT-8 cells or trace inhibitors carried over from cell maintenance media may be responsible for this. On Plate (v), amplification efficiency was > 120 for all standards. This high efficiency in the freely suspended oocyst standards and their equivalent diluted DNA template in addition to the cell culture standards may be a consequence of proteinase K inhibition carried over in this particular oocyst dilution series. Efficiency was not reduced in the equivalent DNA template dilutions on Plate (v) (Fig. 4c) as would be expected, but this is likely a consequence of no DNA signal present in the lowest two dilutions (resulting in only two data points on the standard curve), which is possibly due to the presence of these inhibitors.

**Fig. 10:**
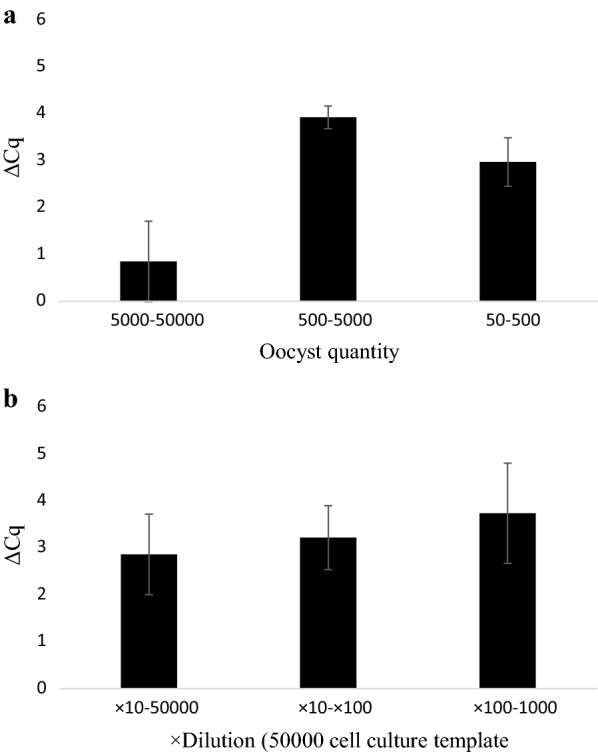
Change in Cq-values (ΔCq) (Plate (vii)) between serial dilutions of *Cryptosporidium parvum* oocysts in inoculated into cell-culture (5 × 10^4^–50) (**a**) and 10-fold dilutions of 5 × 10^4^ (×10, ×100 and ×1000) (**b**). ΔCq calculated by subtracting the Cq-value of the lower concentration from the immediate higher concentration

It has been shown that *C. parvum* does not appear to multiply in a linear fashion in cell culture [15] and differing oocyst seeding density possibly affects parasite growth rates because of competition between parasites in the same well. In addition to polymerase inhibition, these factors may be responsible for lower ΔCq at high concentrations in cell culture standards. To what extent each of these factors is responsible for reduced linearity remains to be determined. These factors will also affect the template dilution of cell culture standards. If, for example parasite growth is less proliferative at higher seeding concentrations, dilutions of this template will not necessarily reflect DNA content obtained from lower inoculates. Indeed, diluted DNA template from 5 × 10^4^ oocyst cell culture standards on Plate (vii) yielded significantly less DNA at ×10 and ×1000 dilutions (the equivalent of 5 × 10^3^ and 50 oocysts, respectively) than oocysts incubated in cell culture (Figs. 9, 10).

Although there is increased nucleic DNA content in cell culture derived standards, most likely an effect of asexual multiplication of the parasite in host cells, their suitability as standards over those derived from pure oocyst suspensions is not clear. The higher DNA content in cell culture derived standards will more accurately reflect DNA content in cell culture assays. However, the reduced linearity as a result of more variable ΔCq between oocyst concentrations (due to either oocyst seeding variation inherent in cell culture inoculations, polymerase inhibitors or different oocyst seeding densities onto the monolayer) will make it more difficult to interpret relative parasite inhibition based on the standard curve. Some studies have utilised oocyst suspensions as standards for *Cryptosporidium* cultured in cells or in axenic culture [22, 23]. As disinfection, development and drug screening studies are less dependent upon the absolute quantification of the parasite, rather, relative quantification, provided DNA standards remain the same across assays, it is possible to assess these aforementioned parameters [21, 41].

## Conclusions

To facilitate inter-laboratory comparisons of qPCR results, standardised protocols are required including procedures for generating standard DNA templates if precise data are needed. Although numerous studies have reported DNA content from cell cultured *C. parvum* previously and these have been compared to *in vivo* models [24], in the present study, the clear suitability of template dilutions of stock oocysts for use as standards was established, despite a higher DNA content in cell culture templates at equivalent concentrations. Our results indicate that overall there was a consensus in the values obtained from the same templates analysed on different plates. The potential loss of cell viability at the highest seeding densities could suggest the use of inoculates < 10^5^ would be appropriate for cell culture assays. However, this should be considered in light of the propensity of increased variation and ΔCq in cell culture at lower oocyst inoculates. As only relative growth inhibition is required for many assays involving *C. parvum* it would appear the pure oocyst suspensions or diluted DNA templates may represent ideal standards at present. Since cell culture derived standards will more accurately reflect actual DNA content from parasite grown in cell culture however, there is a clear incentive for prospective study to focus on the mechanisms explaining this reduced linearity.

## Supplementary information

**Additional file 1: Table S1.** Cq-values of quantitative PCR Plate (i). *Cryptosporidium parvum* oocysts inoculated onto HCT-8 cell monolayer plate A. Two technical repeats for each well (biological repeat). *Abbreviations*: TR, technical repeat; BR, biological repeat; SD, standard deviation.

**Additional file 2: Table S2.** Cq-values of quantitative PCR Plate (ii). *Cryptosporidium parvum* oocysts inoculated onto HCT-8 cell monolayer plate B. Two technical repeats for each well (biological repeat). *Abbreviations*: TR, technical repeat; BR, biological repeat; SD, standard deviation.

**Additional file 3: Table S3.** Cq-values of quantitative PCR Plate (iii). Pure *C. parvum* oocyst serial dilution A and B. Two technical repeats for each Eppendorf (biological repeat). *Abbreviations*: TR, technical repeat; SD, standard deviation.

**Additional file 4: Table S4.** Cq-values of quantitative PCR Plate (iv). Pure *C. parvum* oocyst serial dilution A and B and equivalent template dilution A and B. Two technical repeats for each Eppendorf (biological repeat). *Abbreviations*: TR, technical repeat; SD, standard deviation.

## Data Availability

The datasets used and/or analyzed during the present study are available from the corresponding author upon reasonable request.

## Abbreviations

PCR: polymerase chain reaction
qPCR: quantitative polymerase chain reaction
HCT-8: human ileocecal adenocarcinoma
Cq: quantification cycle
ddPCR: digital droplet polymerase chain reaction
PBS: phosphate-buffered saline
DAPI/PI: 4ʹ,6-diamidino-2phenylindole/propidium iodide
ECACC: European collection of authenticated cell cultures
FBS: fetal bovine serum
EDTA: ethylenediaminetetraacetic acid
DMSO: dimethyl sulfoxide
WST-1: water-soluble tetrazolium assay
OD: optical density
ATL: animal tissue lysis
NTC: non-template controls
dsDNA: double stranded DNA
ΔCq: delta Cq (change in Cq-values)
RFU: relative fluorescence units
