# Supplementary material for: Assessment of differences between DNA content of cell-cultured and freely suspended oocysts of Cryptosporidium parvum and their suitability as DNA standards in qPCR
Source: Parasit Vectors. 2019 Dec 19;12:596. doi: 10.1186/s13071-019-3851-7 (PMC6923971; doi:10.1186/s13071-019-3851-7)
Supplement: Supplementary file 1 — Additional file 1: Table S1. Cq-values of quantitative PCR Plate (i). Cryptosporidium parvum oocysts inoculated onto HCT-8 cell monolayer plate A. Two technical repeats for each well (biological repeat). Abbreviations: TR, technical repeat; BR, biological repeat; SD, standard deviation. [file 13071_2019_3851_MOESM1_ESM.docx]

**Additional file 1: Table S1.** Cq-values of quantitative PCR Plate (i). *Cryptosporidium parvum* oocysts inoculated onto HCT-8 cell monolayer plate A. Two technical repeats for each well (biological repeat). *Abbreviations*: TR, technical repeat; BR, biological repeat; SD, standard deviation.

| **Oocyst Inoculate** |  | **100000** | **Mean T.R. (±S.D.)** | **Mean B.R. (±S.D.)** | **10000** | **Mean T.R. (±S.D.)** | **Mean B.R. (±S.D.)** | **1000** | **Mean T.R. (±S.D.)** | **Mean B.R. (±S.D.)** | **100** | **Mean T.R. (±S.D.)** | **Mean B.R. ± (S.D.)** | **10** | **Mean T.R. (±S.D.)** | **Mean B.R. (±S.D.)** | **1** | **Mean T.R. (±S.D.)** | **Mean B.R. (±S.D.)** |
| --- | --- | --- | --- | --- | --- | --- | --- | --- | --- | --- | --- | --- | --- | --- | --- | --- | --- | --- | --- |
| **Baseline threshold 12.56 RFU**  **(Auto-threshold)** | Technical repeat |  |  |  |  |  |  |  |  |  |  |  |  |  |  |  |  |  |  |
| **Well 1** | 1 | 30.38 | 30.04 (±0.49) | 28.76 (±0.81) | 30.04 | 29.15 (±1.27) | 29.69 (±0.38) | 35.24 | 35.32 (±0.11) | 33.45 (±1.09) | 38.39 | 37.45 (±1.34) | 36.5 (±0.66) | 44.33 | 41.71 (±3.71) | 41.28 (±2.54) | 40.2 | 41.58 (±1.94) | 42.66 (±1.19) |
|  | 2 | 29.69 |  |  | 28.25 |  |  | 35.39 |  |  | 36.5 |  |  | 39.09 |  |  | 42.95 |  |  |
| **Well 2** | 1 | 28.05 | 28.07 (±0.03) |  | 30 | 30.07 (±0.09) |  | 33.09 | 33.05 (±0.06) |  | 37.13 | 36.2 (±1.32) |  | N/A | N/A |  | 40.73 | 41.77 (±1.46) |  |
|  | 2 | 28.09 |  |  | 30.13 |  |  | 33.01 |  |  | 35.27 |  |  | 41.88 |  |  | N/A |  |  |
| **Well 3** | 1 | 27.45 | 28.13 (±0.96) |  | 30.08 | 30.02 (±0.08) |  | 32.92 | 32.73 (±0.28) |  | 37.61 | 36.55 (±1.51) |  | 37.42 | 37.6 (±0.25) |  | 43.78 | N/A |  |
|  | 2 | 28.81 |  |  | 29.96 |  |  | 32.53 |  |  | 35.48 |  |  | 37.78 |  |  | N/A |  |  |
| **Well 4** | 1 | 29.01 | N/A |  | 29.61 | 29.55 (0.08) |  | 33.07 | 33.47 (±0.56) |  | 35.18 | 35.64 (±0.64) |  | N/A | N/A |  | 43.45 | 44.11 (±0.93) |  |
|  | 2 | N/A |  |  | 29.49 |  |  | 33.86 |  |  | 36..09 |  |  | 44.64 |  |  | 44.76 |  |  |
| **Well 5** | 1 | 29.09 | 28.55 (±0.77( |  | 30.21 | 29.66 (±0.76) |  | 31.59 | 32.68 (±1.53) |  | 36.32 | 36.67 (±0.49) |  | 37.1 | 40.56 (±4.89) |  | N/A | N/A |  |
|  | 2 | 28 |  |  | 29.14 |  |  | 33.76 |  |  | 37.03 |  |  | 44.01 |  |  | 42.06 |  |  |
|  | Technical repeat | **Inactivated oocysts (100000)** | **Mean T.R. (±S.D.)** | **Mean B.R. (±S.D.)** | **Blank** | **Mean T.R. (±S.D.)** | **Mean B.R. (±S.D.)** |  |  |  |  |  |  |  |  |  |  |  |  |
| **Well 1** | 1 | N/A | N/A | 36.34 (±0.56) | 43.89 | 43.73 (±0.23) | 41.8 (±2) |  |  |  |  |  |  |  |  |  |  |  |  |
|  | 2 | 37.27 |  |  | 43.57 |  |  |  |  |  |  |  |  |  |  |  |  |  |  |
| **Well 2** | 1 | 35.4 | 35.95 (±0.78) |  | 42.28 | 43.32 (±1.46) |  |  |  |  |  |  |  |  |  |  |  |  |  |
|  | 2 | 36.5 |  |  | 44.35 |  |  |  |  |  |  |  |  |  |  |  |  |  |  |
| **Well 3** | 1 | 36.02 | 36.23 (±0.29) |  | N/A | N/A |  |  |  |  |  |  |  |  |  |  |  |  |  |
|  | 2 | 36.43 |  |  | N/A |  |  |  |  |  |  |  |  |  |  |  |  |  |  |
| **Well 4** | 1 | 36.23 | 35.9 (±0.47) |  | 39.94 | N/A |  |  |  |  |  |  |  |  |  |  |  |  |  |
|  | 2 | 35.57 |  |  | N/A |  |  |  |  |  |  |  |  |  |  |  |  |  |  |
| **Well 5** | 1 | 36.31 | 36.36 (±0.07) |  | N/A | N/A |  |  |  |  |  |  |  |  |  |  |  |  |  |
|  | 2 | 36.41 |  |  | 40.21 |  |  |  |  |  |  |  |  |  |  |  |  |  |  |

Cq-values of quantitative PCR Plate (i). *Cryptosporidium parvum* oocysts inoculated onto HCT-8 cell monolayer plate A. Two technical repeats for each well (biological repeat). *Abbreviations*: TR, technical repeat; BR, biological repeat; SD, standard deviation.
