# Supplementary material for: Assessment of differences between DNA content of cell-cultured and freely suspended oocysts of Cryptosporidium parvum and their suitability as DNA standards in qPCR
Source: Parasit Vectors. 2019 Dec 19;12:596. doi: 10.1186/s13071-019-3851-7 (PMC6923971; doi:10.1186/s13071-019-3851-7)
Supplement: Supplementary file 2 — Additional file 2: Table S2. Cq-values of quantitative PCR Plate (ii). Cryptosporidium parvum oocysts inoculated onto HCT-8 cell monolayer plate B. Two technical repeats for each well (biological repeat). Abbreviations: TR, technical repeat; BR, biological repeat; SD, standard deviation. [file 13071_2019_3851_MOESM2_ESM.docx]

**Additional file 2: Table S2.** Cq-values of quantitative PCR Plate (ii). *Cryptosporidium parvum* oocysts inoculated onto HCT-8 cell monolayer plate B. Two technical repeats for each well (biological repeat). *Abbreviations*: TR, technical repeat; BR, biological repeat; SD, standard deviation.

| **Oocyst Inoculate** |  | **100000** | **Mean T.R. (±S.D.)** | **Mean B.R. (±S.D.)** | **10000** | **Mean T.R. (±S.D.)** | **Mean B.R. (±S.D.)** |  | **1000** | **Mean T.R. (±S.D.)** | **Mean B.R. (±S.D.)** | **100** | **Mean T.R. (±S.D.)** | **Mean B.R. ± (S.D.)** | **10** | **Mean T.R. (±S.D.)** | **Mean B.R. (±S.D.)** | **1** | **Mean T.R. (±S.D.)** | **Mean B.R. (±S.D.)** |
| --- | --- | --- | --- | --- | --- | --- | --- | --- | --- | --- | --- | --- | --- | --- | --- | --- | --- | --- | --- | --- |
| **Baseline threshold 8.00 RFU** | Technical repeat |  |  |  |  |  |  |  |  |  |  |  |  |  |  |  |  |  |  |  |
| **Well 1** | 1 | 29.34 | 29.3 (±0.06) | 28.91 (±0.85) | 31.13 | 31.74 (±0.78) | 30.43 (±0.88) |  | 36.39 | 36.03 (±0.52) | 34.73 (±0.74) | 37.29 | 37.27 (±0.04) | 37.39 (±0.6) | N/A | N/A | N/A | 41.33 | N/A | 43.27 (±1.26) |
|  | 2 | 29.26 |  |  | 32.29 |  |  |  | 35.66 |  |  | 37.24 |  |  | 40.07 |  |  | N/A |  |  |
| **Well 2** | 1 | 29.59 | 29.86 (±0.37) |  | 29.78 | 29.47 (±0.45) |  |  | 34.37 | 34.35 (±0.03) |  | 37.45 | 37.86 (±0.58) |  | 41.5 | N/A |  | 43.04 | NA |  |
|  | 2 | 30.12 |  |  | 29.15 |  |  |  | 34.33 |  |  | 38.27 |  |  | N/A |  |  | N/A |  |  |
| **Well 3** | 1 | N/A | N/A |  | 31.19 | 30.78 (±0.58) |  |  | 35.01 | 34.63 (± 0.54) |  | 36.59 | 36.68 (±0.13) |  | N/A | N/A |  | 44.09 | 44.09 (±0) |  |
|  | 2 | N/A |  |  | 30.37 |  |  |  | 34.25 |  |  | 36.77 |  |  | N/A |  |  | 44.09 |  |  |
| Well 4 | 1 | 27.65 | 28.13 (±0.34) |  | 30.34 | 30.3 (±0.06) |  |  | 34.05 | 34.34 (±0.410 |  | N/A | N/A |  | N/A | N/A |  | 43.89 | 44.08 (±0.27) |  |
|  | 2 | 28.13 |  |  | 30.26 |  |  |  | 34.63 |  |  | 38.14 |  |  | N/A |  |  | 44.27 |  |  |
| **Well 5** | 1 | 28.33 | 28.62 (±0.41) |  | 30.29 | 29.86 (±0.62) |  |  | 34.37 | 34.29 (±0.12) |  | 37.04 | 37.02 (±0.04) |  | 41.82 | 41.82 (±41.1) |  | 40.54 | 41.88 (±1.9) |  |
|  | 2 | 28.91 |  |  | 29.42 |  |  |  | 34.2 |  |  | 36.99 |  |  | 40.29 |  |  | 43.22 |  |  |
|  | Technical repeat | **Inactivated oocysts (100000)** | **Mean T.R. (±S.D.)** | **Mean B.R. (±S.D.)** | **Blank** | **Mean T.R. (±S.D.)** | **Mean B.R. (±S.D.)** |  |  |  |  |  |  |  |  |  |  |  |  |  |
| **Well 1** | 1 | 36.84 | 37.37 (±0.75) | 37.33 (±0.72) | 41.34 | 43.09 (±234) | 43.35 (±0.52) |  |  |  |  |  |  |  |  |  |  |  |  |  |
|  | 2 | 37.9 |  |  | 44.72 |  |  |  |  |  |  |  |  |  |  |  |  |  |  |  |
| **Well 2** | 1 | 38.42 | 38.41 (±0.01) |  | 43.67 | N/A |  |  |  |  |  |  |  |  |  |  |  |  |  |  |
|  | 2 | 38.4 |  |  | N/A |  |  |  |  |  |  |  |  |  |  |  |  |  |  |  |
| **Well 3** | 1 | 37.45 | N/A |  | N/A | N/A |  |  |  |  |  |  |  |  |  |  |  |  |  |  |
|  | 2 | N/A |  |  | N/A |  |  |  |  |  |  |  |  |  |  |  |  |  |  |  |
| Well 4 | 1 | 37.26 | 36.98 (±0.4) |  | N/A | N/A |  |  |  |  |  |  |  |  |  |  |  |  |  |  |
|  | 2 | 36.69 |  |  | 42.8 |  |  |  |  |  |  |  |  |  |  |  |  |  |  |  |
| **Well 5** | 1 | 36.67 | 36.44 (±0.33) |  | 43.91 | N/A |  |  |  |  |  |  |  |  |  |  |  |  |  |  |
|  | 2 | 36.21 |  |  | N/A |  |  |  |  |  |  |  |  |  |  |  |  |  |  |  |

Cq-values of quantitative PCR Plate (ii). *Cryptosporidium parvum* oocysts inoculated onto HCT-8 cell monolayer plate B. Two technical repeats for each well (biological repeat). *Abbreviations*: TR, technical repeat; BR, biological repeat; SD, standard deviation.
