# Supplementary material for: Assessment of differences between DNA content of cell-cultured and freely suspended oocysts of Cryptosporidium parvum and their suitability as DNA standards in qPCR
Source: Parasit Vectors. 2019 Dec 19;12:596. doi: 10.1186/s13071-019-3851-7 (PMC6923971; doi:10.1186/s13071-019-3851-7)
Supplement: Supplementary file 3 — Additional file 3: Table S3. Cq-values of quantitative PCR Plate (iii). Pure C. parvum oocyst serial dilution A and B. Two technical repeats for each Eppendorf (biological repeat). Abbreviations: TR, technical repeat; SD, standard deviation. [file 13071_2019_3851_MOESM3_ESM.docx]

**Additional file 3: Table S3.** Cq-values of quantitative PCR Plate (iii). Pure *C. parvum* oocyst serial dilution A and B. Two technical repeats for each Eppendorf (biological repeat). *Abbreviations*: TR, technical repeat; SD, standard deviation.

| **Oocyst Quantity** |  | **1000000** | **Mean T.R. (S.D.)** | **100000** | **Mean T.R. (S.D.)** | **10000** | **Mean T.R. (S.D.)** | **1000** | **Mean T.R. (S.D.)** | **100** | **Mean T.R. (S.D.)** | **100** | **Mean T.R. (S.D.)** | **10** | **Mean T.R. (S.D.)** | **1** | **Mean T.R. (S.D.)** | **MQ** | **Mean T.R. (S.D.)** |
| --- | --- | --- | --- | --- | --- | --- | --- | --- | --- | --- | --- | --- | --- | --- | --- | --- | --- | --- | --- |
| **Baseline threshold 20 RFU** | Technical repeat |  |  |  |  |  |  |  |  |  |  |  |  |  |  |  |  |  |  |
| **Serial dilution A** | 1 | 29.87 | 29.45 (±0.42) | 31.59 | 31.34 (±0.25) | 34.33 | 34.61 (±0.28) | N/A | NA | N/A | N/A | N/A | N/A | N/A | N/A | N/A | N/A | N/A | N/A |
|  | 2 | 29.03 |  | 31.09 |  | 34.89 |  | 38 |  | N/A | N/A | N/A | N/A | N/A | N/A | N/A | N/A | N/A | N/A |
| **Serial dilution B** | 1 | 29.16 | 28.74 (±42) | 30.44 | 30.29 (±0.16) | 33.64 | 33.86 (±0.22) | 37.84 | 37.01 (±0.75) | N/A | N/A | N/A | N/A | N/A | N/A | N/A | N/A | N/A | N/A |
|  | 2 | 28.32 |  | 30.13 |  | 34.07 |  | 36.35 |  | N/A | N/A | N/A | N/A | N/A | N/A | N/A | N/A | N/A | N/A |

Cq-values of quantitative PCR Plate (iii). Pure *C. parvum* oocyst serial dilution A and B. Two technical repeats for each Eppendorf (biological repeat). *Abbreviations*: TR, technical repeat; SD, standard deviation.
