# Supplementary material for: Assessment of differences between DNA content of cell-cultured and freely suspended oocysts of Cryptosporidium parvum and their suitability as DNA standards in qPCR
Source: Parasit Vectors. 2019 Dec 19;12:596. doi: 10.1186/s13071-019-3851-7 (PMC6923971; doi:10.1186/s13071-019-3851-7)
Supplement: Supplementary file 4 — Additional file 4: Table S4. Cq-values of quantitative PCR Plate (iv). Pure C. parvum oocyst serial dilution A and B and equivalent template dilution A and B. Two technical repeats for each Eppendorf (biological repeat). Abbreviations: TR, technical repeat; SD, standard deviation. [file 13071_2019_3851_MOESM4_ESM.docx]

**Additional file 4: Table S4.** Cq-values of quantitative PCR Plate (iv). Pure *C. parvum* oocyst serial dilution A and B and equivalent template dilution A and B. Two technical repeats for each Eppendorf (biological repeat). *Abbreviations*: TR, technical repeat; SD, standard deviation.

| **Oocyst Quantity** |  | **1000000** | **Mean T.R. (S.D.)** | **100000** | **Mean T.R. (S.D.)** | **10000** | **Mean T.R. (S.D.)** | **1000** | **Mean T.R. (S.D.)** | **100** | **Mean T.R. (S.D.)** | **10** | **Mean T.R. (S.D.)** | **1** | **Mean T.R. (S.D.)** | **MQ** | **Mean T.R. (S.D.)** |
| --- | --- | --- | --- | --- | --- | --- | --- | --- | --- | --- | --- | --- | --- | --- | --- | --- | --- |
| **Baseline threshold 12 RFU** | Technical repeat |  |  |  |  |  |  |  |  |  |  |  |  |  |  |  |  |
| **Serial dilution A** | 1 | 28.22 | 28.79 (±0.8) | 30.55 | 31.09 (±0.25) | 34.27 | 34.24 (±0.03) | 37.42 | 38.17 (±0.75) | 42.84 | 43.34 (±0.5) | N/A | N/A | N/A | N/A | N/A | N/A |
|  | 2 | 29.35 |  | 31.63 |  | 34.21 |  | 38.92 |  | 43.84 |  | N/A | N/A | N/A | N/A | N/A | N/A |
|  |  |  |  | **×10** |  | **×100** |  | **×1000** |  | **×10000** |  | **×100000** |  | **×1000000** |  |  |  |
| **Template dilution A** | 1 |  |  |  |  |  |  |  |  |  |  |  |  |  |  |  |  |
|  | 2 |  |  | 31.38 | 31.04 (±0.35) | 34.61 | 34.09 (±0.53) | N/A | N/A | 41.16 | 40.82 (±0.34) | N/A | N/A | N/A | N/A |  |  |
| **Serial dilution B** |  |  |  | 30.69 |  | 33.56 |  | 38.11 |  | 40.48 |  | N/A |  |  | N/A |  |  |
|  | 1 | 28.07 | 27.62 (±0.67) | 30.2 | 30.35 (±30.35) | 33.4 | 33.64 (±0.15) | 37.39 | 37.95 (±0.56) | 41.16 | 40.82 (±0.34) | N/A | N/A | N/A | N/A | N/A | N/A |
|  | 2 | 27.17 |  | 30.5 |  | 33.88 |  | 38.5 |  | 40.48 |  | N/A | N/A | N/A | N/A | N/A |  |
|  |  |  |  | **×10** |  | **×100** |  | **×1000** |  | **×10000** |  | **×100000** |  | **×1000000** |  |  |  |
| **Template dilution B** | 1 |  |  | 30.36 | 30.52 (±0.17) | 35.15 | 35.11 (±0.04) | 38.11 | 38.19 (±0.08) | 41.37 | N/A | N/A | N/A | N/A | N/A | N/A | N/A |
|  | 2 |  |  | 30.69 |  | 35.07 |  | 38.26 |  | N/A |  | N/A |  | N/A |  | N/A |  |

Cq-values of quantitative PCR Plate (iv). Pure *C. parvum* oocyst serial dilution A and B and equivalent template dilution A and B. Two technical repeats for each Eppendorf (biological repeat). *Abbreviations*: TR, technical repeat; SD, standard deviation.
